# Key considerations, target product profiles, and research gaps in the application of infrared spectroscopy and artificial intelligence for malaria surveillance and diagnosis

**DOI:** 10.1186/s12936-023-04780-3

**Published:** 2023-11-10

**Authors:** Issa H. Mshani, Doreen J. Siria, Emmanuel P. Mwanga, Bazoumana BD. Sow, Roger Sanou, Mercy Opiyo, Maggy T. Sikulu-Lord, Heather M. Ferguson, Abdoulaye Diabate, Klaas Wynne, Mario González-Jiménez, Francesco Baldini, Simon A. Babayan, Fredros Okumu

**Affiliations:** 1 Ifakara Health Institute, Environmental Health, and Ecological Sciences Department, Morogoro, United Republic of Tanzania; 2 School of Biodiversity, One Health and Veterinary Medicine, University of Glasgow, Glasgow, UK; 3 School of Life Sciences and Biotechnology, Nelson Mandela African Institution of Science and Technology, Arusha, United Republic of Tanzania; 4 School of Public Health, The University of the Witwatersrand, Park Town, South Africa; 5 Department of Medical Biology and Public Health, Institut de Recherche en Sciences de la Santé (IRSS), Bobo-Dioulasso, Burkina Faso; 6 Centro de Investigação em Saúde de Manhiça (CISM), Maputo, Mozambique; 7 Faculty of Science, School of the Environment, The University of Queensland, Brisbane, QLD Australia; 8 School of Chemistry, The University of Glasgow, Glasgow, G12 8QQ UK; 9 Malaria Elimination Initiative (MEI), Institute for Global Health Sciences, University of California, San Francisco, USA

**Keywords:** Malaria surveillance, Infrared spectroscopy, Artificial intelligence, Machine learning, Deep learning, Target product profiles

## Abstract

Studies on the applications of infrared (IR) spectroscopy and machine learning (ML) in public health have increased greatly in recent years. These technologies show enormous potential for measuring key parameters of malaria, a disease that still causes about 250 million cases and 620,000 deaths, annually. Multiple studies have demonstrated that the combination of IR spectroscopy and machine learning (ML) can yield accurate predictions of epidemiologically relevant parameters of malaria in both laboratory and field surveys. Proven applications now include determining the age, species, and blood-feeding histories of mosquito vectors as well as detecting malaria parasite infections in both humans and mosquitoes. As the World Health Organization encourages malaria-endemic countries to improve their surveillance-response strategies, it is crucial to consider whether IR and ML techniques are likely to meet the relevant feasibility and cost-effectiveness requirements—and how best they can be deployed. This paper reviews current applications of IR spectroscopy and ML approaches for investigating malaria indicators in both field surveys and laboratory settings, and identifies key research gaps relevant to these applications. Additionally, the article suggests initial target product profiles (TPPs) that should be considered when developing or testing these technologies for use in low-income settings.

## Background

Effective control of malaria requires an in-depth understanding of its transmission. This entails estimating parasitological, entomological and epidemiological parameters in respective communities [1]. Specific activities may include detecting malaria infections in humans, estimating mosquito survival following deployment of interventions, identifying malaria-infected mosquitoes, and characterizing the human populations at risk [1]. As countries move towards malaria elimination in line with the strategic goals of the World Health Organization (WHO) [2], there is a need to develop simple, low-cost and scalable methods for assessing key entomological and parasitological indicators of malaria and for monitoring the impact of interventions [1, 3–6].

Proper management of suspected malaria cases requires confirmation through quality-assured laboratory tests [2, 7]. These tests may also include quantifying the number of asexual malaria parasites in blood samples to determine the severity of the infection, or identifying carriers through the detection of *Plasmodium* gametocytes [8, 9]. In population surveys, malaria prevalence can be estimated through various methods, such as observing malaria parasites under a microscope, using rapid diagnostic tests (RDTs) to detect parasite-derived proteins and by-products, or detecting parasite nucleic acid sequences through polymerase chain reactions (PCR) [4, 10]. These tools have greatly improved the diagnosis of malaria, guided effective case management, and enhanced the evaluation of key interventions.

In terms of entomological indicators, female *Anopheles* can transmit malaria only if they live long enough to pick up the infective stages of *Plasmodium*, and thereafter incubate those parasites until they mature into the infectious sporozoite stage. This process usually takes 10–14 days, but can be slower depending on climatic conditions [11, 12]. Proportions of female mosquitoes that are old enough to transmit malaria can, therefore, be used to estimate vectorial capacity (number of mosquito infective bites produced by a single malaria case) and assess the performance of vector control methods, such as insecticide-treated nets (ITNs) and indoor residual spray (IRS) [13–16].

The primary measure of malaria transmission intensity, the entomological inoculation rate (EIR), is calculated as the product of the human biting rates (number of bites per unit of time) and the proportion of mosquitoes infected with *Plasmodium* sporozoites. Estimating EIR requires detailed assessments of *Anopheles* biting rates, typically through mosquito trapping, and the proportion of female *Anopheles* that carry infective *Plasmodium* sporozoites, typically through enzyme-linked immunosorbent assays (ELISA) or PCR [17, 18]. In addition to these core entomological metrics, other measures can be used to estimate the natural survival and transmission potential of *Anopheles* populations. These may include ovarian dissections to assess parous proportions, analysis of vertebrate blood meals to estimate the proportion of mosquitoes biting humans, and estimation of the proportion of mosquitoes biting indoors and outdoors [1, 19].

Although these strategies for monitoring malaria transmission have contributed to progress against the disease [20], there are still considerable obstacles related to operational costs, performance accuracies, scalability, and human resource requirements [5, 6, 21]. In order to align with global priorities for malaria elimination, further advancements in both entomological and parasitological surveillance are just as important as the need for new drugs, vaccines, or vector control approaches [2, 6].

A recent advancement in malaria monitoring is the use of infrared (IR) spectroscopy in combination with machine learning (ML) techniques to assess key indicators of malaria. These indicators include the chronological age of mosquitoes (e.g. number of days post emergence) [22–25], blood-feeding histories of malaria vectors [26], *Plasmodium* infections in human blood [27–29] or mosquitoes [30], and identification of malaria vector species [22]. In this technique, biological samples are scanned with infrared radiation, and the energy absorbed by the covalent bonds in the target specimen causes its molecules to vibrate. An infrared spectrum generates information about the molecules that absorb the radiation and their intensity of absorption [31]. Despite the subtle biochemical differences between specimens with different biological traits, ML algorithms can disentangle these spectral changes and map them to specific phenotypes [27, 31, 32]. Together, IR and ML-based systems constitute robust, easy-to-use, reagent-free, non-invasive and low-cost approaches, making them attractive in low-income settings [22, 24, 26]. As a result, there has been a significant increase in the number of studies evaluating or validating these techniques for monitoring vector-borne diseases [23, 33, 34].

To ensure maximum benefits going forward, it is important to identify existing gaps and the essential and desirable characteristics that should be met for these technologies to be effectively integrated into routine malaria control programmes. The aim of this article is to review existing IR spectroscopy and ML applications for malaria surveillance and diagnostics, to identify gaps for field use, and to outline a target product profiles for such technologies to be suitable in low-income settings.

## Current methods for measuring malaria transmission

### Parasitological methods

The most common method for parasitological assessment of malaria is light microscopy, which is standard practice in many laboratories and relies on direct observations of malaria parasites on thick or thin smears of blood [35–37]. Although light microscopy is accessible even in low-resource settings, it requires highly experienced personnel and can generally detect only parasite densities above 50 parasites/μl of blood with an overall sensitivity of between 50 and 500 parasites/μl [38, 39]. The method may, therefore, miss individuals with low parasitaemia levels or asymptomatic carriers [37, 38], and may perform poorly in low-transmission settings [42]. The diagnostic accuracy might also be compromised by poor preparation of thick or thin blood smears and visual identification [43, 44].

Another common approach is the use of malaria rapid diagnostic tests (RDTs), which have revolutionized malaria investigations in both clinical settings and community surveys due to their low-cost and promptness [45, 46]. Moreover, they do not require highly-trained or experienced personnel to perform or interpret the tests, and can be used even in hard-to-reach areas, and by community healthcare workers [45, 47]. Most RDTs target the parasite antigen, histidine-rich protein II (HRP-2), which is abundant in *P. falciparum* infected red blood cells [38, 48]. Some RDTs also target glycolytic enzymes, such as *Plasmodium* aldolase and *Plasmodium* lactate dehydrogenase (pLDH) antigens, and can detect non-*falciparum* malaria parasites, such as *Plasmodium ovale, Plasmodium malariae* and* Plasmodium vivax* [38, 49].

The main disadvantages of RDTs include the lack of quantitative information and poor performance in asymptomatic cases or low-level parasitaemia, such as those with parasitaemia levels below 100 parasites/μl [4, 47]. In addition, genetic mutations of the HRP-2 genes, which are spreading around the world, also compromise the sensitivity of RDTs [51–58]. These gene deletions, which have so far been detected in nearly 40 countries [59], make the malaria parasites undetectable by the HRP-2 based RDTs even when the patients are severely ill. The WHO currently recommends that countries should withdraw these specific RDTs if more than 5% of malaria infections have HRP-2 mutations [59].

**Fig. 1:**
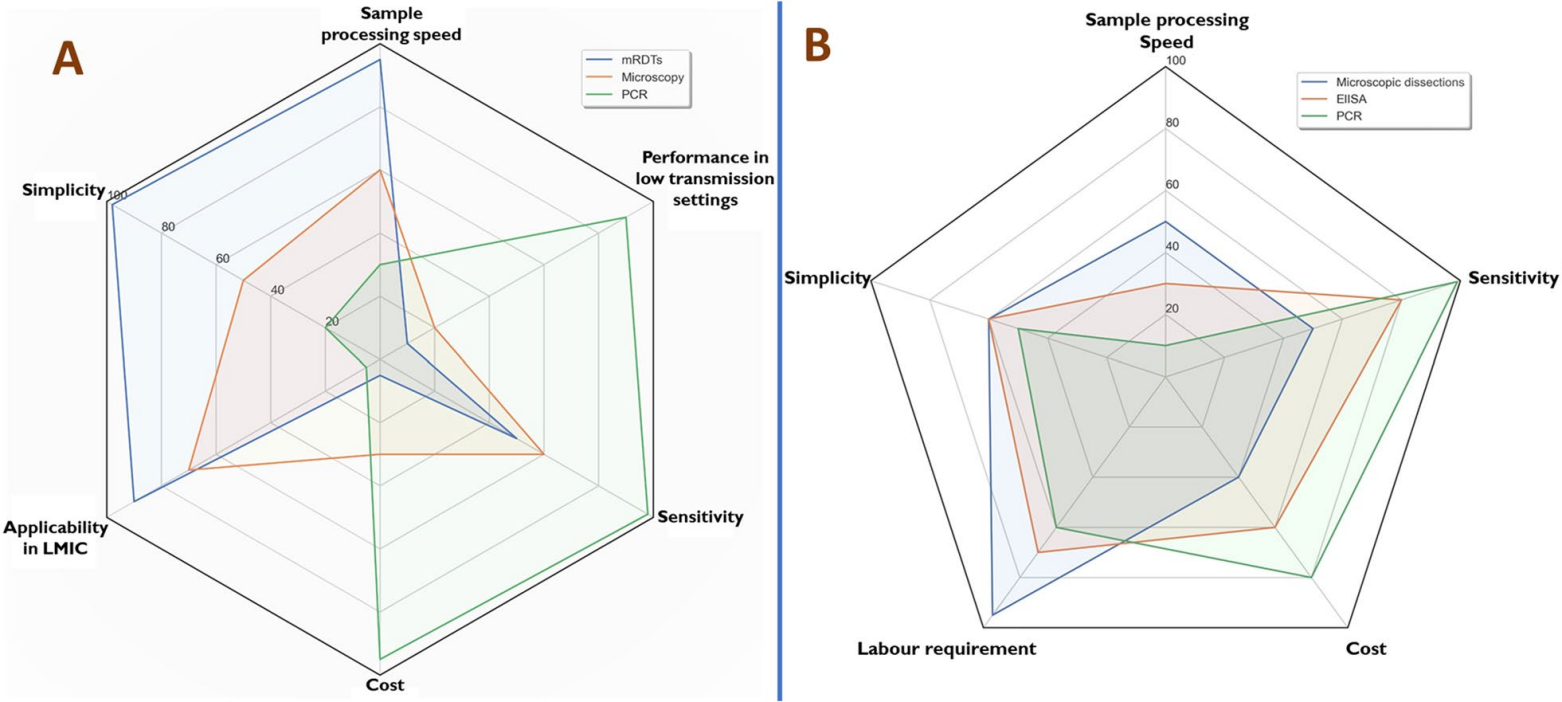
Applicability, strengths, and weaknesses of current methods used to measure key malaria indicators. Panel **A** compares the three most common methods for parasitological assessment [51, 64–72] while panel **B** compares the three main methods for entomological assessment [73–83], on a proportion score of 1–100. These scores are based on expert opinion of the authors of this article

Nucleic acid-based diagnostics, such as PCR, have the highest sensitivity but are often unaffordable in most malaria-endemic settings and are, therefore, rarely used [60–62]. PCR can detect parasitaemia as low as 1–5 parasites/μl of blood, but is used mostly in research settings because of its high cost and the need for specialized facilities and personnel [38, 62, 63]. Epidemiological surveys have also demonstrated that PCR assays can be used to identify areas with unusually high malaria transmission [37, 42]. Moreover, one analysis of methods for detecting malaria hotspots in coastal Kenya concluded that PCR was the most appropriate for mapping asymptomatic cases once overall prevalence had dropped significantly [42]. Lastly, PCR also provides detailed information on *Plasmodium* species based on the small subunit 18S rRNA or circumsporozoite protein genes, and can also detect mixed infections [38, 62]. Unfortunately, as summarized in Fig. 1A, the techniques require highly-skilled labour, expensive equipment and reagents, making them untenable for regular use in places with poor supply of laboratory materials [4].

#### Entomological methods

The WHO has outlined several entomological indicators that malaria programs may consider for monitoring transmission dynamics, guiding the selection or deployment of control strategies, and evaluating the control efforts [1, 7]. These include: (i) mosquito blood-feeding histories, biting frequencies, and resting behaviours, (ii) vector species presence and densities (iii) insecticide resistance status, (iv) proportion of mosquitoes with *Plasmodium* sporozoites, and (v) larval habitat profiles [1, 20]. The indicators may be differently prioritized depending on the local capabilities, malaria epidemiological profiles, financial constraints, and prevailing control strategies in the respective countries. However, the most central ones are biting rate, mosquito density and EIR.

Entomological surveys involve different sampling methods, after which the collected mosquitoes are sorted by taxa and physiological features. Adult female mosquitoes are frequently dissected for analysis of their internal organs (e.g. gut, reproductive systems and salivary glands) or retained for other laboratory analyses [19]. Sex and species are initially sorted based on the exterior morphology of the mosquitoes using taxonomic keys [84, 85]; but the indistinguishable members of species complexes, such as *Anopheles gambiae *sensu lato (*s*.*l*.) and *Anopheles. funestus group* require further distinction by PCR [86–88]. As summarized in Fig. 1B, these methods are time-consuming, expensive, require specialized training, and are not always readily available locally [89].

Depending on the research goals, additional laboratory tests may be performed on the collected mosquitoes. These can include ELISA tests to detect malaria parasite proteins or PCR tests to find *Plasmodium* sporozoites in the heads and thoraces of female *Anopheles* mosquitoes [83, 90, 91]. Additionally, examination of the stomach contents of the mosquitoes, using ELISA [92] or PCR [93], can be performed to identify the vertebrate sources of mosquito blood meals as required to determine their preference for biting humans compared to other animals. The age of field-collected female mosquitoes is generally determined by dissection to examine changes in their ovaries; with age here being estimated in terms of reproductive history (e.g. whether parous or not, and if parous how many gonotrophic cycles have been completed) rather than in terms of chronological age (e.g. number of days post emergence) [19, 77, 94, 95]. Estimates of physiological age derived from these dissection methods are used to approximate chronological age based on fixed assumption of the number of days required to complete a gonotrophic cycle in the field [76, 96]. Finally, a series of bio-efficacy and molecular assays to determine the resistance of the mosquitoes to insecticides, to inform appropriate insecticidal interventions can also be done [97].

Entomological monitoring is complex and costly, and as a result, only a small number of malaria-endemic countries can monitor all the recommended entomological parameters on a large scale [20, 21, 98]. A recent analysis of vector surveillance programmes in malaria-endemic countries found that countries with the highest burden have far less surveillance capacity than countries nearing elimination [20]. Overall, most countries are not well-equipped to establish effective surveillance systems with the minimum essential data necessary to detect changes and adjust public health responses.

## Applications of infrared spectroscopy and machine learning for parasitological and entomological surveys of malaria

The attributes of mosquitoes or human body tissues can be studied by analysing their infrared (IR) spectral signatures. These signatures contain complex biochemical information represented by absorbance intensities at different wavenumbers. Near Infrared (NIR) Spectroscopy specifically measures absorption by vibrational overtones, or vibrations that are excited from the ground state to the second or third energy level, in the 14,000–4000 cm^-1^ range. On the other hand, Mid-Infrared (MIR) spectroscopy measures absorption by fundamental vibrations of molecular bonds in the 4000–400 cm^-1^ range, which allows for more direct quantification of functional chemical groups present in substances, such as chitin, protein, or wax in the samples of interest [22, 33].

Once the samples have been scanned, ML algorithms can be used to analyse the infrared spectral data and identify specific entomological and parasitological parameters [23–25, 28, 99]. Additional techniques may be used to remove errors and improve the accuracy of the analysis, such as transfer learning [100]. These algorithms can be used to determine features such as mosquito age, species identity, infection status, and blood meal types. Studies have shown that combining IR spectroscopy with machine learning (IR-ML) can provide accurate predictions and estimates of various transmission indicators [33]. For example, this approach has been used to classify malaria-transmitting mosquitoes by chronological age or number of gonotrophic cycles [22, 23], making it useful for studying the effects of vector control on mosquito populations. The potential of IR-ML techniques for measuring malaria transmission should be evaluated based on factors such as robustness, speed, validity, infrastructure needs, scalability, costs, and cost-effectiveness; and should only be adopted if they address the challenges of conventional methods.

The cost of IR spectroscopy equipment used for malaria research can vary greatly. Hand-held versions of NIR or MIR spectrometer can cost as little as $2000 [101], while desktop versions of these spectrometers range from $30,000 to $60,000 [27, 99]. Most equipment is durable for regular laboratory or field use and requires minimal maintenance. No additional reagents are needed for operation, except standard low-cost maintenance, such as providing desiccants to limit humidity effects.

### Parasitological surveys

The use of infrared spectroscopy (IR) for diseases screening and diagnostic purposes has been demonstrated in various branches of medicine, including the histopathological screening of breast cancers and the prediction of infections (e.g. enterococci), leukaemia, Alzheimer’s, epilepsy, skin carcinoma, brain oedema, and diabetes [102, 103]. An increasing number of studies are also using IR-ML techniques for parasitological screening [33].

In one study in Tanzania, dried blood spots were scanned using an attenuated total reflection-Fourier Transform Infrared spectrometer (ATR-FTIR) and a logistic regression model was trained using the resulting MIR spectra [27]. This approach achieved 92% accuracy relative to PCR in identifying individuals infected with *P. falciparum*, and 85% accuracy for detecting mixed infections such as those carrying both *P. falciparum* and *P. ovale* [27], which is also common in some parts of Tanzania [104]. Another study used synchrotron MIR Fourier Transform Infrared spectroscopy coupled with artificial neural networks (ANN) to achieve a prediction accuracy of 100% for distinguishing between different stages of the cultured *P. falciparum*, *i*.*e*., rings, trophozoites and schizonts [29]. Additionally, support vector machine algorithms fitted with MIR spectra from an ATR-FT spectrometer were used to classify infected and uninfected individuals with a sensitivity of 92% and specificity of 97% [28]. Recently, it has been shown that the NIR absorption peaks of malaria parasites can be used for non-invasive detection of malaria infection through human skin using miniaturized hand-held spectrometers [101].

### Entomological surveys

Using off-the-shelf hardware, both NIR and MIR spectroscopy can be used to analyse large numbers of mosquito samples at a relatively low cost compared to traditional methods [22, 33, 94, 105]. Studies have demonstrated direct applications to predict mosquito chronological (e.g. number of days post emergence) and physiological age classes (e.g. whether parous or not), blood-feeding histories and species identity [22, 24–26, 105, 106]. They have also been used to detect mosquito endosymbionts, such as *Wolbachia* [107, 108], and mosquito-borne pathogens, such as Zika [34, 109] and malaria [110, 111]. Scientists have attempted to validate these laboratory findings in the field settings but so far, there has been success in only a small number of studies [23, 25, 112].

There has been a particular interest in evaluating the potential of IR-ML systems for mapping the demographic characteristics of wild mosquito populations and using this to evaluate the performance of vector control interventions, or monitoring transmission risk. Following multiple successes in combining IR spectroscopy and ML for age-grading laboratory and semi-field vector populations [22, 105], field studies are now underway to validate the potential of this tool in malaria-endemic communities. It is expected that these ongoing efforts will deliver a scalable and operationally relevant IR-ML system that integrates off-the-shelf hardware and open-source software to simplify the technologies. Ultimately, IR-ML based approaches will be most desirable only if they constitute a simple set of routine activities that can be performed by researchers and National Malaria Control Programme (NMCP) staff.

Despite advancements in using IR-ML for malaria surveillance, there remain several challenges that might hinder its full potential. In entomological surveillance, existing algorithms show up to 99% accuracy with laboratory-reared mosquitoes, but this drops significantly in field data, due to variances in mosquito body compositions from dietary, genetic, and environmental factors [22, 23, 33, 112, 113]. Fortunately, recent strides using Convolutional Neural Networks (CNN) have improved accuracy across diverse datasets, with CNNs achieving over 90% accuracy in laboratory and field assessments on specific mosquito species [24]. Transfer learning is also being explored to enhance algorithm generalizability in real-world settings [100]. Additionally, logistical hurdles akin to those faced by existing surveillance methods exist, particularly in hardware maintenance and supply [20]. Moreover, unlike other diagnostic methods with built-in verification, IR-ML lacks this feature [42, 114], indicating a vital area for future research to ensure reliable operations and address these identified gaps. The sections below will discuss these gaps and potential solutions in detail.

## Considerations for research and development of IR-ML approaches for malaria survey and diagnostics

As the WHO encourages malaria-endemic countries to scale up effective surveillance-response strategies for malaria, an important question that remains is what elements should be considered when developing or evaluating new approaches such as IR spectroscopy and ML. Moreover, while IR-ML technologies have the potential to aid in malaria surveillance, several gaps in research and development must be addressed to optimize their utility. This section identifies key research and development gaps in the applications of these technologies for malaria surveys in low-income settings. It further proposes a target product profile (TPP) consisting of both the essential characteristics and desirable characteristics that could improve its uptake, performance, and cost-effectiveness.

## Research gaps to be addressed

Table 1 provides a summary of the key research gaps for the IR-ML applications relevant to malaria. For each of these gaps, additional details are provided below.

**Table 1:** A summary of key research questions and potential research agenda for IR-ML applications relevant to malaria surveys and diagnostics

R&D gaps	Descriptions and examples	References
Incomplete understanding of the IR spectroscopic signals relative to specific biological traits	There is an insufficient understanding of the IR spectroscopic signals (vibrational absorption bands/wavelengths) and their association with biological traits such as parasite infections, age, species, and blood meals	[22, 26, 27, 118]
Inadequate field validation of the IR-ML approaches	There is insufficient field validation of the performance of IR-ML methods for assessing important entomological and parasitological indicators	[22, 23, 28, 120]
Gaps in machine learning frameworks for the IR spectroscopy analysis	There is a need for studies to identify optimal ML objectives such as computational efficiency, prediction accuracy, and model generalizability. This might entail one or a combination of the many existing unsupervised and supervised algorithms	[22, 23, 28, 123]
Unknown detection thresholds	There has not been sufficient demonstration of the limits of detection of IR-ML techniques for detecting malaria infections in human or mosquito samples	[32, 118, 123]
Uncertain granularity of discretized biological outcomes	It is uncertain which method of classifying mosquito age is the best. For example, comparing classification by specific days (1, 2, 3, 4 days) to using longer ranges of days (1, 3, 5, 7 days) or grouping days into ranges (1–5, 5–7, 7–10 days) is unclear	[22, 105]
Resolving overlap and interactions between signals	For biological indicators such as blood meal identification, the possibilities of detecting mixed blood sources remain unknown, and how long after feeding, the blood can still be detected	[22, 105]
Lack of evidence from different epidemiological profiles or settings	There is a need to demonstrate the performance of the IR-ML techniques for detecting malaria parasites in areas with varying epidemiological strata- with low to high transmission or prevalence, and in conditions with varying parasite densities	[27, 28]
Gaps related to hardware and software for IR and ML	There are limited off-the-shelf portable tools that are completely ready for applications in malaria surveys and diagnostics in both laboratory and field settings	[26, 28, 118]
Need to standardize sample-handling procedures	There is currently no standardized protocol for sample handling when using IR-ML methods for malaria surveys and diagnostics	[113, 119, 130]

*Gap 1: Need for a greater understanding of the biochemical and physicochemical basis of the IR signals relevant for malaria surveys and diagnosis.*

IR spectral absorption intensities are determined by the chemical bonds within chitin, protein, and wax, which are the three most abundant components of the mosquito cuticle. Recent research has shown that these signals can be used to infer the age and species of mosquitoes [22, 23], as well as distinguish between *Plasmodium*-infected and uninfected human blood [27].

In the case of parasitological observations, it is apparent that the most dominant spectral features that influence ML model predictions are found in the fingerprint region (1730–883 cm^−1^), where most of the signal from biological samples is expected [27]. The breakdown of haemoglobin into haemozoin crystals may also show up in the IR spectra and can help detect infections in blood samples [27, 115]. The interpretation of IR spectra should, therefore, take into account the sample type and characteristics, and in the case of composite sample types such as parasite-infected blood, considerations on how the parasite interacts with and alters the biochemical makeup of the host tissues should be factored in. For example, changes in the carbohydrate regions at 1144, 1101 and 1085 cm^−1^ may be associated with differential glucose levels in infected red blood cells, since *Plasmodium* parasites metabolize glucose faster than normal cells [32, 116]. The presence of these direct and indirect signals of infection raises the possibility of non-specific detection and false positive diagnoses, which should be mitigated with carefully chosen controls when training machine learning (ML) models.

Given the many different applications of IR-ML for investigating malaria indicators, a generalized framework is essential and should be derived from an updated understanding of the bio-chemical and physico-chemical features of the samples.

*Gap 2: Need to validate the performance of the IR-ML in different field settings and laboratories*

So far, most of the successes in the application of IR-ML have been with laboratory specimens, but it has been difficult to apply these laboratory-trained algorithms to field-collected specimens due to environmental, laboratory, and genetic sources of variation, and/or limited training data. Only a small number of studies have achieved this with partial success [23, 25, 112]. This challenge is compounded by the limited generalizability of many existing ML models. Efforts towards field validation should be integrated with those that seek to improve the generalizability of the models, using more diverse datasets with greater genetic and environmental variability.

Field validation is also essential for IR-based parasitological surveys, as algorithms trained using spectral data from laboratory parasite cultures may not be applicable to other settings, in part due to different immunological and physiological profiles [117–119]. Consequently, early validation of IR-ML for parasitological surveillance is essential and the training data should capture representative signals associated with immunological and genetic composition from multiple populations [27, 120]. For clinical applications, determining the true effectiveness of these techniques may also require large-scale clinical studies.

*Gap 3: Need for appropriate ML-frameworks that achieve maximum predictive accuracies with minimal computational power.*

While IR-ML approaches for assessing malaria indicators can achieve high accuracies, there are many differences between the analytical methods and algorithms used. Currently, there is no consensus on the best ML frameworks for spectral analysis, either supervised or unsupervised. Ideally, the best framework would be that which provides minimum computational needs while also achieving accurate and generalizable predictions of the target traits.

Initially, multivariate statistics, including partial least squares (PLS) and principal component analysis (PCA) were the most widely used [32, 116, 119, 121, 122]. More recently, IR spectroscopy coupled with different ML classifiers has been used to link the signals of IR biochemical bands to specific biological traits [33]. A general approach is to compare and select from multiple model types. Diverse ML algorithms, such as support vector machines (SVMs), Random Forests (RFs), K-nearest neighbours (KNNs), Naive Bayes (NBs), Gradient Boosts (XGBs), and Multilayer Perceptrons have been tested for their ability to decipher IR spectra associated with malaria indicators [24, 25, 27–29].

Unsupervised learning is often utilized in spectra pre-processing to decrease dimensionality or cluster dominant features before algorithm training [23, 28, 29], but additional statistical techniques can be added to improve generalization. For example, unsupervised PCA was used to reduce the dimensionality of the data set, and an ANN was trained on the pre-processed data to accurately predict malaria parasite stages [29]. Moreover, transfer learning and dimensionality reduction techniques like PCA and t-SNE (t-distributed stochastic neighbour embedding) can significantly reduce computational power while maintaining robust accuracy in models [100].

*Gap 4: Need to understand the malaria parasite detection thresholds for the IR-ML systems.*

As conventional methods have a low likelihood of detecting malaria infections with low parasitaemia [61], it is necessary to understand the lower limits of detection (LLOD) for any novel diagnostic and screening tools. Unfortunately, only a small number of studies have examined such thresholds for IR-based malaria detection. One study which used serially diluted parasites grown in vitro demonstrated that ATR-FTIR data could be used to identify and quantify parasite densities as low as < 1 parasite/μl [32]. Other research has shown that NIR spectroscopy coupled with PCA and PLS can detect up to 0.5 parasite/μl and quantify up to 50 parasite/μl parasites in isolated RBCs [118].

Other studies have also shown that using wider spectral ranges, e.g. combining the UV, Visual and IR spectra, can accurately detect and measure malaria without the need for complex preservation methods [118, 123]. Nevertheless, most studies that established the LLODs of the IR did not use ML as the framework for interpreting parasite signals in IR spectra. There has been no investigation of threshold detections for malaria parasites using IR-ML approaches in field settings. Future research should, therefore, establish absolute or the relative LLOD of the IR-ML techniques in both the point-of-care applications and population surveys.

*Gap 5: Understanding the performance and validity of the IR-ML techniques in settings with varying epidemiological profiles.*

Malaria screening methods perform differently in settings with varying transmission intensities and parasitaemia. The most sensitive markers of malaria infections at low transmission intensities tend to be nucleic acids or antibodies to *P. falciparum* [124]. One study in coastal Kenya that compared RDTs, light microscopy, and PCR showed that malaria transmission hotspots detectable by PCR overlapped with those detectable by microscopy at a moderate transmission setting but not low transmission settings [42]. Elsewhere, the effectiveness of RDTs and microscopy was greatest in regions with high malaria transmission or in the presence of high parasitaemia [125]. These tests can however miss many infections in low-transmission regions, where microscopy-negative individuals may still contribute 20–50% of infections sustaining transmission [126].

According to the WHO-backed “High Burden, High Impact” malaria strategy [127], endemic countries are encouraged to implement sub-nationally tailored plans that differentially address high and low malaria burdens. This requires sensitive, high-throughput, and fast screening tools for malaria with comparable validity across transmission settings [2, 5]. Unfortunately, the performance of new tools such as IR-ML has not been compared across settings. Researchers have been able to identify malaria-positive MIR spectra with models trained on pooled data from both low and high transmission settings or from high transmissions only, but not across strata [27, 28]. Additionally, the positive predictive value (ability to predict true positive cases), and negative predictive value (ability to predict true negative cases) should be clarified. One study in Tanzania estimated the positive and negative predictive values of IR-ML at 92.8 and 91.7%, respectively for detecting malaria infections field-collected dried blood spots relative to PCR [27], but this study had only a small number of samples. Future studies should include broad demonstrations of the performance of IR-ML approaches in different epidemiological strata.

*Gap 6: The need for essential human resource training in malaria-endemic countries.*

The implementation of effective malaria surveillance in endemic countries is hindered by inadequacies of trained personnel and facilities. A global survey found that only 8% of malaria-endemic countries had sufficient capacity for vector surveillance and nearly 50% had no capacity to implement core interventions [21]. To effectively implement IR-ML based surveillance at the country level, two forms of training are necessary: one for potential users, including researchers and malaria surveillance officers, and one for higher-level experts capable of tasks such as manipulating infrared and machine learning systems and creating new classification algorithms. Countries may also implement periodic refresher training to boost human resource capabilities [128].

To ensure sustainability and effectiveness, a comprehensive and strategic training plan involving the development of IR-ML training guidelines and partnerships with research and academic institutions is necessary.

*Gap 7: Need to select the most appropriate hardware and software platforms.*

Selecting suitable hardware and software platforms is crucial for enhancing the scalability of IR-ML systems for malaria surveys and diagnostics. A mix of hardware, such as sample collection devices and spectrometers, and software, such as spectral filters and ML models, is necessary. Portable devices are available for field surveys [22, 23, 26], but they are mostly in clinical or research laboratory settings. To implement them on a large scale, spectrometers with hardware systems designed for areas with limited electricity access, such as solar-powered or battery-powered spectrometers, may be necessary. Other options may include the miniaturized IR spectrometers, such as those recently used to detect and quantify malaria parasites in RBCs [118] and for non-invasive parasite detection via the skin of human beings [101].

Spectral data must also be easily interpretable for non-expert users in remote settings. This may require deploying trained algorithms on cloud-based platforms and designing user-friendly interfaces that work with simple internet connectivity. Systems based on mobile phone applications [28] or web interfaces [129] are already being tested, and can be enhanced to remain functional even under limited internet connectivity in remote settings. Lastly, the availability of relevant source codes (preferably via code-sharing platforms such as GitHub) and training in their use should also be ensured.

*Gap 8: Need to standardize sample-handling procedures.*

Standardized protocols for sample handling are needed to ensure the comparability of findings and to make IR-ML techniques more widely applicable in parasitological and entomological assessments. Unfortunately, little effort has been devoted to determining the optimal methods for storing and preserving samples for IR-ML investigations. For entomological studies, some protocols have indicated using chloroform to kill specimens and storing them in silica gel for 2–3 days before scanning [22, 26], and also that NIR spectroscopy performs well when samples are stored by either desiccants, RNAlater, or refrigeration [130]. Separately, a study using MIR spectroscopy and ML demonstrated the crucial need for standardized handling (storage or preservation) for both training and validation samples [113].

Proper sample storage and preservation is also essential for reducing spectral noise and preserving the biochemical composition of the specimens. For example, the use of anticoagulant materials can significantly affect model performance when using dried RBCs compared to the wet RBCs or whole blood when scanned using ATR-FTIR spectroscopy [119]. While some of these challenges can be addressed by statistical approaches, e.g. transfer learning [100], optimal performance requires a level of standardization in methods for handling different sample types destined for IR-ML analysis.

### Target product characteristics of the IR and ML approaches

To guide further development and evaluation of the IR-ML based approaches for parasitological and entomological investigations of malaria, this article proposes an initial outline of key characteristics that should be met. This target product profile (TPP) consolidates the current thoughts and expertise of the authors as experts and early adopters of the application of this technology for malaria surveys. However, this TPP is subject to future modifications and should be considered as a preliminary version. To satisfy the global strategies for malaria monitoring, the draft describes the necessary and desirable qualities of emerging IR and ML-based techniques for use in both field surveys and clinical settings (Tables 2, 3)***.***

**Table 2:** Proposed Target Product Profile (TPP) for an IR-ML based system for parasitological surveillance of malaria (focusing on detecting parasites in vertebrate host)

Characteristics		Parasitological surveillance			
		Passive case detection in clinical settings (symptomatic cases)		Active case detection in field screening (asymptomatic cases)	
		Essential characteristics	Desired characteristics	Essential characteristics	Desired characteristics
Scope	Intended use settings and contexts	As point-of-care test in malaria control settings	As point of care test in elimination and control settings	Malaria screening in moderate to high transmission settings	Can be used in low, moderate and high transmission settings
	Implementation level	District-level health facilities, other centralized facilities & research facilities	Can be used in peripheral health facilities, e.g., dispensaries, health posts & health centres	Used for surveys in research facilities	Routine parasitological surveys in district or regional hospitals
	Types of spectrometers and durability	Bench-top units that require minimum electricity, and can operate 5 years or more with minimal maintenance	Off –the-shelf portable units with long-life battery; can be solar-powered; can operate for up to 10 years with minimal maintenance	Bench-top units that require minimum electricity, and can operate 5 years or more with minimal maintenance	Off –the-shelf portable units with long-life battery; can be solar-powered; can operate for up to 10 years with minimal maintenance
Technical performance	Sensitivity relative to conventional methods [50, 125, 132]	Can detect > 95% of positive *P. falciparum* cases in symptomatic individuals relative to RDT	Can detect > 99% of positive *P. falciparum* cases in symptomatic individuals relative to RDT or microscopy	Detects > 95% of positive *P. falciparum* cases in moderate to high transmission areas relative to microscopy or RDT or PCR	Detects > 95% of positive *P. falciparum* cases in low, moderate or high transmission areas relative to microscopy or PCR
	Specificity relative to current tests [50, 125, 132]	Can identify > 95% of malaria-negative cases in febrile individuals relative to RDT	Can identify > 99% of malaria-negative cases in febrile individuals relative to RDT or microscopy	Can identify > 95% of malaria-negative cases in areas with moderate to high transmission relative to microscopy or RDT or PCR	Can identify > 99% of malaria-negative cases in low, moderate to high transmission areas relative to microscopy or PCR
	Resolution and accuracy of predictions	Achieves at least 95% accuracy compared to RDT or microscopy	Performance equivalent to microscopy or RDTs) in detecting *P. falciparum* & other malaria parasites	Has > 95% accuracy compared to RDT or microscopy in moderate high transmission sites	Performance matches PCR in detecting malaria parasites under low—high transmission
Technical performance	Lower limit of parasite detection (LOD)	50–100 parasites/μl of blood; equivalent to microscopy & RDTs in respective settings	Less than 50 parasite/μl of blood; better than microscopy & RDTs in respective settings	50–100 parasites/μl of blood; equivalent to microscopy and RDTs in respective settings	Less than 50 parasite/μl of blood; better than microscopy & RDTs; equivalent to PCR
	Temperature and humidity stability	Stable in ambient temperatures; can withstand occasional increases to 30 °C for short periods	Stable in ambient temperatures; withstands occasional increases to 35 °C for short periods	Stable in ambient temperatures and can withstand increases to 35 °C for short periods and varied humidity	Stable in ambient temperatures and can withstand increases to 45 °C for short periods and varied humidity
Operational aspects	Equipment & maintenance costs	Less than $ 30,000 per spectrometer; lasts > 5 years; costs < $100/yr to maintain	Less than $ 2000 per portable spectrometer; lasts up to 10 years; Costs < $50/Yr to maintain	Less than $ 30,000 per spectrometer; lasts > 5 years; costs < $100/yr to maintain	Less than $ 2000 per portable spectrometer; lasts up to 10 years; Costs < $50/Yr to maintain
	Sample handling costs	Costs < $0.1 per test	Costs < $0.01per test	Costs < $0.1 per test	Costs < $0.01per test
	Test duration	< 5 min	< 1 min	< 5 min	< 1 min
	Sample preservation and storage	Requires freezing, desiccants, RNAlater Uses must be less than 30 days old	Can use any preservation method Samples can be older than 6 months as long as preserved fresh	Freezing, desiccants or RNAlater; Samples must be less than 30 days old	Any preservation method; Samples can be older than 6 months as long as preserved fresh
Operational aspects	Human resources: Skills & training	Requires minimal training on sample handling, scanning & data interpretation	No more than 30 min training needed to use, conduct the tests and read results	Requires minimal training on sample handling, scanning & data interpretation	No more than 30 min training needed to use, conduct the tests and read results
	Type of sample	Wet or dry blood samples; presented as glass slides, on filter papers or as blood drops	Both blood and non-blood samples (saliva, urine, sweat or other samples collected non-invasively e.g. over the skin)	Wet or dry blood samples; presented as glass slides, on filter papers or as blood drops	Both blood and non-blood samples (saliva, urine, sweat or other samples collected non-invasively e.g. via skin)
	Reagents	No reagents needed except for cleaning the instruments or sample collection	No reagents needed except for cleaning the instruments or sample collection	No reagents needed except for cleaning the instruments or sample collection	No reagents needed except for cleaning the instruments or sample collection

**Table 3:** Proposed Target Product Profile (TPP) for an IR-ML based system for entomological surveillance of malaria

Characteristics		Entomological surveillance	
		Essential characteristics	Desired characteristics
Scope	Intended use	Mosquito identification; mosquito age-grading, detection of *Plasmodium*-infected mosquitoes and blood meal identity	One-stop platform for most desired entomological indicators; species IDs, age grading, blood meal identifications, infection detection and assessment of insecticide resistance status i.e. ability to predict resistance phenotype as per standard bioassays
	Implementation level	Can be used in research laboratories and training centres	Used for research and routine surveillance services in laboratory and field settings
	Types of spectrometers and durability	Bench-top units that require minimum electricity, and can operate 5 years or more with minimal maintenance	Off –the-shelf portable units with long-life battery; can be solar-powered; can operate for up to 10 years with minimal maintenance
Technical performance	Sensitivity relative to current methods [17, 18]	*Plasmodium* sporozoites**:** Sensitivity of > 90% with reference to ELISA	*Plasmodium* sporozoites**:** Sensitivity of > 90% with reference to PCR
	Specificity relative to current methods [17, 18, 83]	*Plasmodium* sporozoites**:** Specificity of > 90% with reference to ELISA, Microscopy or PCR	*Plasmodium* sporozoites**:** Specificity of > 90% with reference to PCR
	Resolution of predictions	*Mosquitoes age*: can classify young (e.g. 1–6 days) vs old (e.g. > 10 days) mosquitoes, with > 90% accuracy *Species identification***:** Can distinguish between members of a species complex (e.g. *An. gambiae* vs. *An. arabiensis*) with > 90% accuracy *Mosquito blood meals***:** Can distinguish between human blood meal from any other vertebrate blood meals, with > 90% accuracy	*Mosquitoes age***:** Can classify mosquito ages chronologically at 2-day resolution (e.g. 1, 3, 5 days etc.) with > 90% accuracy *Species***:** Can identify all major malaria vectors (*An. gambiae, An. arabiensis, An. coluzzii* and *An. funestus*) with > 90% accuracy *Blood meal:* Can identify most vertebrate blood meals (i.e. human, cattle, goat, chicken, dog etc.) even if the meals are mixed, and with > 90% accuracy
	Lower limit of parasite detection	*Plasmodium* sporozoites**:** has performance equivalent to ELISA or PCR	*Plasmodium* sporozoites**:** has performance equivalent to PCR
	Temperature and humidity stability	Functions and can be stored in ambient temperature; withstands frequent temperature rises to 35 °C for a long periods under diverse humidity	Functions and can be stored in ambient temperature; withstands frequent temperature rises to 45 °C for a long periods under diverse humidity
Operational aspects	Equipment & maintenance costs	Less than $ 30,000 per spectrometer; lasts > 5 years; costs < $100/yr to maintain	Less than $ 2000 per portable spectrometer; lasts > 5 yrs; Costs < $50/Yr to maintain
	Sample handling costs	Costs < $0.1 per test	Costs < $0.01per test
	Test duration	< 5 min	< 1 min
	Sample preservation and storage	Requires freezing, desiccants, RNAlater Uses must be less than 30 days old	Can use any preservation method Samples can be older than 6 months as long as preserved fresh
Operational aspects	Human resources: Skills & training	Requires minimal training on sample handling, scanning & data/results interpretation	No more than 30 min training needed to use, conduct the tests and read results
	Type of sample	Can analyse dried mosquito body parts	Can analyse either fresh or dried mosquito body parts
	Reagents	No reagents needed except for cleaning the instruments or sample collection	No reagents needed except for cleaning the instruments or sample collection

Different TPPs have previously been proposed for future vector surveillance tools [95] and malaria diagnostic tools [131]. The article complements these by proposing relevant attributes for IR-ML techniques including both parasitological (Table 2) and entomological measures (Table 3). The proposed profile presents both the core characteristics, which are the minimum basic requirements for a functional system, as well as other desirable characteristics that could further improve the capabilities, scalability, and cost-effectiveness of this technology.

## Conclusion

The combination of infrared spectroscopy and machine learning is being considered a promising new method for predicting or estimating various entomological and parasitological indicators of malaria. The IR-ML platforms have the added advantage of being simple to use, reagent-free, high-throughput, low-cost, and applicable in rural and remote settings. As malaria-endemic countries seek to enhance their surveillance-response strategies to achieve elimination targets, an important question is how IR-ML-based approaches can complement ongoing processes and be integrated into routine surveillance. This paper has reviewed existing IR and ML applications and their gaps for malaria surveys and parasite screening; with provision of initial suggestions on target product profiles (TPPs) for such technologies in low-income settings. The TPPs outline both essential and desirable attributes to guide further development. The article also outline key research and development gaps that should be addressed in the short and medium term, including the need for field validation, determination of minimum detection threshold, capacity development and training in user countries, assessment of the validity of the tests in different epidemiological strata, and work on robust hardware and software to enable expanded use.

## Data Availability

Not applicable.

## Abbreviations

IR: Infrared spectroscopy
ML: Machine learning
AI: Artificial intelligence
TPPs: Target product profiles
ANN: Artificial neural networks
SVM: Support vector machine
ATR: Attenuated total reflectance
LR: Logistic regression
NIRs: Near infrared spectroscopy
MIRs: Mid Infrared Spectroscopy
